# Supplementary material for: Advances in reversible covalent kinase inhibitors
Source: Med Res Rev. 2024 Sep 17;45(2):629–53. doi: 10.1002/med.22084 (PMC11796325; doi:10.1002/med.22084)
Supplement: Supplementary file 1 — Supporting information. [file MED-45-629-s001.docx]

Supporting Information

**Advances in Reversible Covalent Kinase Inhibitors**

**Zheng Zhao*^1,2*^* and Philip E. Bourne*^1,2^****

*^1.^*School of Data Science and *^2.^*Department of Biomedical Engineering, University of Virginia, Charlottesville, Virginia 22904, United States of America

*Corresponding author

Email: [zz7r@virginia.edu](mailto:zz7r@virginia.edu) (Z.Z.) and [peb6a@virginia.edu](mailto:peb6a@virginia.edu) (P.E.B)

**Table S1.** The collected RCKIs.

| Index | Kinase groups | Kinase targets | Name in References | Reactive warheads | Active residues | Ref |
| --- | --- | --- | --- | --- | --- | --- |
| 9-15 | TK | BTK | 1-4; 6-8 | Cyanoacrylamide | C481 | ^1^ |
| 16 | TK | BTK | 9(Phase-III) | Cyanoacrylamide | C481 | ^1^ |
| 17 | TK | BTK | 61 | Chlorofluoroacetamide | C481 | ^2^ |
| 18 | TK | BTK | 9a | Cyanamide | C481 | ^3^ |
| 19 | TK | BTK | 10 | Cyanamide | C481 | ^3^ |
| 20 | TK | BTK | 11a | Cyanamide | C481 | ^3^ |
| 21 | TK | BTK | 20a | Cyanamide | C481 | ^3^ |
| 22 | TK | BTK | 20b | Cyanamide | C481 | ^3^ |
| 23 | TK | BTK | 20c | Cyanamide | C481 | ^3^ |
| 24 | TK | BTK | RC-1 | Cyanoacrylamide | C481 | ^4^ |
| 25 | TK | BTK | RC-2 | Cyanoacrylamide | C481 | ^4^ |
| 26 | TK | BTK | RC-3 | Cyanoacrylamide | C481 | ^4^ |
| 27 | TK | BTK | RC-1 | Cyanoacrylamide | C481 | ^5^ |
| 28-32 | TK | EGFR | 1a,b,c,d,e | Cyanoacrylamide | C797 | ^6^ |
| 33 | TK | JAK3 | 4 | Cyanoacrylamide | C909 | ^7^ |
| 34 | TK | JAK3 | 5 | Cyanoacrylamide | C909 | ^7^ |
| 35 | TK | JAK3 | 31 | Cyanoacrylamide | C909 | ^8^ |
| 36 | TK | JAK3 | 10 | Cyanamide | C909 | ^9^ |
| 37-47 | TK | FGFR1 | 28-38 | Cyanoacrylamide | C486 | ^1^ |
| 48-50 | TK | FGFR4 | 6a,6b,9 | Aldehyde | C552 | ^10^ |
| 51-55 | TK | FGFR4 | 43,57,62,68,85 | Aldehyde | C552 | ^11^ |
| 56 | TK | FGFR4 | 84(Phase-II) | Aldehyde | C552 | ^11^ |
| 57 | AGC | RSK2 | 14 | Cyanoacrylate | C436 | ^12^ |
| 58 | AGC | RSK2 | 15 | Cyanoacrylamide | C436 | ^12^ |
| 59 | AGC | RSK2 | 16 | Cyanoacrylate | C436 | ^12^ |
| 60 | AGC | RSK2 | 11 | Cyanoacrylamide | C436 | ^13^ |
| 61 | AGC | MSK/RSK2 | RMM-46/12 | Cyanoacrylamide | C440/C436 | ^13^ |
| 62 | AGC | RSK2 | 27 | Cyanoacrylamide | C436 | ^8^ |
| 63-64 | Other | PLK1 | 4，10 | Benzothiazole N-Oxide | C67 | ^14^ |
| 65 | Atypical | eEF-2K | MDP | Carbonitrile | C146 | ^15^ |
| 66-68 | TK | ABL1 | 14,12 | Aldehyde Boric acid | K271 | ^16^ |
| PRN473 | TK | BTK | PRN473 (Phase-1) | Cyanoacrylamide | C481 | ^17-18^ |

**References**

1. Bradshaw, J. M.; McFarland, J. M.; Paavilainen, V. O.; Bisconte, A.; Tam, D.; Phan, V. T.; Romanov, S.; Finkle, D.; Shu, J.; Patel, V.; Ton, T.; Li, X.; Loughhead, D. G.; Nunn, P. A.; Karr, D. E.; Gerritsen, M. E.; Funk, J. O.; Owens, T. D.; Verner, E.; Brameld, K. A.; Hill, R. J.; Goldstein, D. M.; Taunton, J., Prolonged and tunable residence time using reversible covalent kinase inhibitors. *Nat. Chem. Biol.* **2015,** *11* (7), 525-531.

2. Shindo, N.; Fuchida, H.; Sato, M.; Watari, K.; Shibata, T.; Kuwata, K.; Miura, C.; Okamoto, K.; Hatsuyama, Y.; Tokunaga, K.; Sakamoto, S.; Morimoto, S.; Abe, Y.; Shiroishi, M.; Caaveiro, J. M. M.; Ueda, T.; Tamura, T.; Matsunaga, N.; Nakao, T.; Koyanagi, S.; Ohdo, S.; Yamaguchi, Y.; Hamachi, I.; Ono, M.; Ojida, A., Selective and reversible modification of kinase cysteines with chlorofluoroacetamides. *Nat. Chem. Biol.* **2019,** *15* (3), 250-258.

3. Schnute, M. E.; Benoit, S. E.; Buchler, I. P.; Caspers, N.; Grapperhaus, M. L.; Han, S.; Hotchandani, R.; Huang, N.; Hughes, R. O.; Juba, B. M.; Kim, K. H.; Liu, E.; McCarthy, E.; Messing, D.; Miyashiro, J. S.; Mohan, S.; O'Connell, T. N.; Ohren, J. F.; Parikh, M. D.; Schmidt, M.; Selness, S. R.; Springer, J. R.; Thanabal, V.; Trujillo, J. I.; Walker, D. P.; Wan, Z. K.; Withka, J. M.; Wittwer, A. J.; Wood, N. L.; Xing, L.; Zapf, C. W.; Douhan, J., 3rd, Aminopyrazole Carboxamide Bruton's Tyrosine Kinase Inhibitors. Irreversible to Reversible Covalent Reactive Group Tuning. *ACS Med. Chem. Lett.* **2019,** *10* (1), 80-85.

4. Gabizon, R.; Shraga, A.; Gehrtz, P.; Livnah, E.; Shorer, Y.; Gurwicz, N.; Avram, L.; Unger, T.; Aharoni, H.; Albeck, S.; Brandis, A.; Shulman, Z.; Katz, B. Z.; Herishanu, Y.; London, N., Efficient Targeted Degradation via Reversible and Irreversible Covalent PROTACs. *J. Am. Chem. Soc.* **2020,** *142* (27), 11734-11742.

5. Guo, W. H.; Qi, X.; Yu, X.; Liu, Y.; Chung, C. I.; Bai, F.; Lin, X.; Lu, D.; Wang, L.; Chen, J.; Su, L. H.; Nomie, K. J.; Li, F.; Wang, M. C.; Shu, X.; Onuchic, J. N.; Woyach, J. A.; Wang, M. L.; Wang, J., Enhancing intracellular accumulation and target engagement of PROTACs with reversible covalent chemistry. *Nat. Commun.* **2020,** *11* (1), 4268.

6. Smith, S.; Keul, M.; Engel, J.; Basu, D.; Eppmann, S.; Rauh, D., Characterization of Covalent-Reversible EGFR Inhibitors. *ACS Omega* **2017,** *2* (4), 1563-1575.

7. Forster, M.; Chaikuad, A.; Bauer, S. M.; Holstein, J.; Robers, M. B.; Corona, C. R.; Gehringer, M.; Pfaffenrot, E.; Ghoreschi, K.; Knapp, S.; Laufer, S. A., Selective JAK3 Inhibitors with a Covalent Reversible Binding Mode Targeting a New Induced Fit Binding Pocket. *Cell Chem. Biol.* **2016,** *23* (11), 1335-1340.

8. London, N.; Miller, R. M.; Krishnan, S.; Uchida, K.; Irwin, J. J.; Eidam, O.; Gibold, L.; Cimermancic, P.; Bonnet, R.; Shoichet, B. K.; Taunton, J., Covalent docking of large libraries for the discovery of chemical probes. *Nat. Chem. Biol.* **2014,** *10* (12), 1066-1072.

9. Casimiro-Garcia, A.; Trujillo, J. I.; Vajdos, F.; Juba, B.; Banker, M. E.; Aulabaugh, A.; Balbo, P.; Bauman, J.; Chrencik, J.; Coe, J. W.; Czerwinski, R.; Dowty, M.; Knafels, J. D.; Kwon, S.; Leung, L.; Liang, S.; Robinson, R. P.; Telliez, J. B.; Unwalla, R.; Yang, X.; Thorarensen, A., Identification of Cyanamide-Based Janus Kinase 3 (JAK3) Covalent Inhibitors. *J. Med. Chem.* **2018,** *61* (23), 10665-10699.

10. Knoepfel, T.; Furet, P.; Mah, R.; Buschmann, N.; Leblanc, C.; Ripoche, S.; Graus-Porta, D.; Wartmann, M.; Galuba, I.; Fairhurst, R. A., 2-Formylpyridyl Ureas as Highly Selective Reversible-Covalent Inhibitors of Fibroblast Growth Factor Receptor 4. *ACS Med. Chem. Lett.* **2018,** *9* (3), 215-220.

11. Fairhurst, R. A.; Knoepfel, T.; Buschmann, N.; Leblanc, C.; Mah, R.; Todorov, M.; Nimsgern, P.; Ripoche, S.; Niklaus, M.; Warin, N.; Luu, V. H.; Madoerin, M.; Wirth, J.; Graus-Porta, D.; Weiss, A.; Kiffe, M.; Wartmann, M.; Kinyamu-Akunda, J.; Sterker, D.; Stamm, C.; Adler, F.; Buhles, A.; Schadt, H.; Couttet, P.; Blank, J.; Galuba, I.; Trappe, J.; Voshol, J.; Ostermann, N.; Zou, C.; Berghausen, J.; Del Rio Espinola, A.; Jahnke, W.; Furet, P., Discovery of Roblitinib (FGF401) as a Reversible-Covalent Inhibitor of the Kinase Activity of Fibroblast Growth Factor Receptor 4. *J. Med. Chem.* **2020,** *63* (21), 12542-12573.

12. Serafimova, I. M.; Pufall, M. A.; Krishnan, S.; Duda, K.; Cohen, M. S.; Maglathlin, R. L.; McFarland, J. M.; Miller, R. M.; Frodin, M.; Taunton, J., Reversible targeting of noncatalytic cysteines with chemically tuned electrophiles. *Nat. Chem. Biol.* **2012,** *8* (5), 471-476.

13. Miller, R. M.; Paavilainen, V. O.; Krishnan, S.; Serafimova, I. M.; Taunton, J., Electrophilic fragment-based design of reversible covalent kinase inhibitors. *J. Am. Chem. Soc.* **2013,** *135* (14), 5298-5301.

14. Pearson, R. J.; Blake, D. G.; Mezna, M.; Fischer, P. M.; Westwood, N. J.; McInnes, C., The Meisenheimer Complex as a Paradigm in Drug Discovery: Reversible Covalent Inhibition through C67 of the ATP Binding Site of PLK1. *Cell Chem. Biol.* **2018,** *25* (9), 1107-1116 e4.

15. Devkota, A. K.; Edupuganti, R.; Yan, C.; Shi, Y.; Jose, J.; Wang, Q.; Kaoud, T. S.; Cho, E. J.; Ren, P.; Dalby, K. N., Reversible covalent inhibition of eEF-2K by carbonitriles. *Chembiochem* **2014,** *15* (16), 2435-2442.

16. Yao, S.; Keller, T.; Foo, K.; Zhang, C.-J.; Yang, W.-Q.; Joy, J.; Kwek, P.; Tee, D.; Liu, B.; Li, R.; Retna, P.; Wee, J.; Poulsen, A.; Baburajendran, N.; Anantharajan, J.; Tang, G.; Quach, D., Strategic Design of Catalytic Lysine-Targeting Reversible Covalent BCR-ABL Inhibitors. *ChemRxiv* **2021**, doi: 10.26434/chemrxiv.13710097.v1.

17. Herter, J. M.; Margraf, A.; Volmering, S.; Correia, B. E.; Bradshaw, J. M.; Bisconte, A.; Hill, R. J.; Langrish, C. L.; Lowell, C. A.; Zarbock, A., PRN473, an inhibitor of Bruton's tyrosine kinase, inhibits neutrophil recruitment via inhibition of macrophage antigen-1 signalling. *Br. J. Pharmacol.* **2018,** *175* (3), 429-439.

18. Owens, T. D.; Brameld, K. A.; Verner, E. J.; Ton, T.; Li, X.; Zhu, J.; Masjedizadeh, M. R.; Bradshaw, J. M.; Hill, R. J.; Tam, D.; Bisconte, A.; Kim, E. O.; Francesco, M.; Xing, Y.; Shu, J.; Karr, D.; LaStant, J.; Finkle, D.; Loewenstein, N.; Haberstock-Debic, H.; Taylor, M. J.; Nunn, P.; Langrish, C. L.; Goldstein, D. M., Discovery of Reversible Covalent Bruton's Tyrosine Kinase Inhibitors PRN473 and PRN1008 (Rilzabrutinib). *J. Med. Chem.* **2022,** *65* (7), 5300-5316.
